# Severe Progression of Scoliosis Beyond Skeletal Maturity in Partial Trisomy 13: A Case Report Expanding the Phenotypic Spectrum

**DOI:** 10.7759/cureus.106286

**Published:** 2026-04-01

**Authors:** Andrea Fabregas, Elyette Lugo, Emily Nice, Vivek P Gupta, Amer F Samdani

**Affiliations:** 1 Orthopedics, Universidad Central del Caribe, Bayamon, USA; 2 Research, Shriners Children's - Philadelphia, Philadelphia, USA; 3 Neurosurgery, Shriners Children's - Philadelphia, Philadelphia, USA

**Keywords:** chromosomal abnormality, partial trisomy 13, patau syndrome, scoliosis, spinal deformity

## Abstract

Partial trisomy 13 (Patau syndrome) is a rare chromosomal disorder with variable survival and a wide spectrum of congenital anomalies. While skeletal abnormalities have been described, scoliosis remains infrequently reported, particularly in adolescents with partial trisomy 13 who survive beyond early childhood. We report the case of an 18-year-old male with partial trisomy 13 due to an unbalanced 13;21 translocation who developed progressive thoracolumbar scoliosis during adolescence. The patient was diagnosed with scoliosis at age 13, measuring 33°, and was initially managed with Boston bracing. Bracing was discontinued after two years following a spontaneous pneumothorax due to concerns regarding pulmonary compromise. Despite skeletal maturity, the spinal deformity progressed to a 98° thoracolumbar curve by age 18. Associated features included hypotonia, microcephaly, and neuromotor delay. Even with the severity of the deformity, the patient remained at baseline functional status, including independent seated mobility and preservation of usual daily function. Following multidisciplinary evaluation and shared decision-making with the family, surgical intervention was deferred because the potential risk of postoperative functional decline was felt to outweigh the expected benefit given his preserved baseline mobility and quality of life. This case represents a rare instance of severe scoliosis progression beyond skeletal maturity in partial trisomy 13, a finding not well described in the existing literature. It highlights the need for close longitudinal surveillance and individualized multidisciplinary management strategies that prioritize functional outcomes and patient-centered goals of care.

## Introduction

Patau syndrome (PS), first described in 1960, is a chromosomal disorder caused by trisomy 13 and is characterized by multisystem congenital anomalies, most commonly affecting the cardiac and nervous systems [1-3]. PS occurs in three forms: complete trisomy 13, mosaic trisomy, and partial trisomy due to unbalanced translocations, the latter accounting for approximately 20% of cases [4-6]. Patients with partial or mosaic trisomy may demonstrate increased survival but exhibit broad phenotypic variability, complicating diagnosis and management. PS is the third most common autosomal trisomy, occurring in approximately one in 5,000 live births, though nearly half of affected pregnancies result in fetal loss [2,3]. Advanced maternal age is a recognized risk factor for trisomy 13, particularly in cases related to meiotic nondisjunction, although the condition can also occur in pregnancies of younger mothers, especially in the setting of translocation-related disease [2,7]. Clinically, PS is associated with craniofacial, neurologic, and skeletal abnormalities, including cleft lip and palate, cerebral malformations, polydactyly, syndactyly, and vertebral anomalies such as segmentation defects and butterfly vertebrae [6,8,9]. Recognition of scoliosis in this population is clinically important because progressive deformity may affect posture, mobility, pulmonary status, and long-term functional outcomes [4,10].

Despite the presence of axial skeletal abnormalities, scoliosis has been infrequently reported in patients with partial trisomy 13. The natural history and optimal management of scoliosis in this population remain poorly defined, particularly in adolescents who survive beyond early childhood. This case report describes severe progression of thoracolumbar scoliosis beyond skeletal maturity in an adolescent with partial trisomy 13, highlighting an unusual deformity pattern and an underrecognized aspect of the phenotypic spectrum.

## Case presentation

An 18-year-old male, diagnosed with partial trisomy 13 at five weeks of age, presented for evaluation of progressive spinal deformity and postural imbalance. He was born via cesarean delivery at 35 weeks’ gestation to a 29-year-old G2P0 mother, with pregnancy complicated by preeclampsia. Neonatal course was significant for respiratory distress, hypotonia, sepsis, hypoglycemia, and anemia, requiring a three-week neonatal intensive care unit admission with mechanical ventilation.

Cytogenetic testing confirmed partial trisomy 13 due to an unbalanced translocation (46, XY, +der (13)t(13;21)(q14;q11.1),-21), with confirmation by fluorescence in situ hybridization. Associated features included agenesis of the corpus callosum, hypotonia, microcephaly, rocker-bottom feet, congenital heart disease, and cryptorchidism.

**Figure 1:**
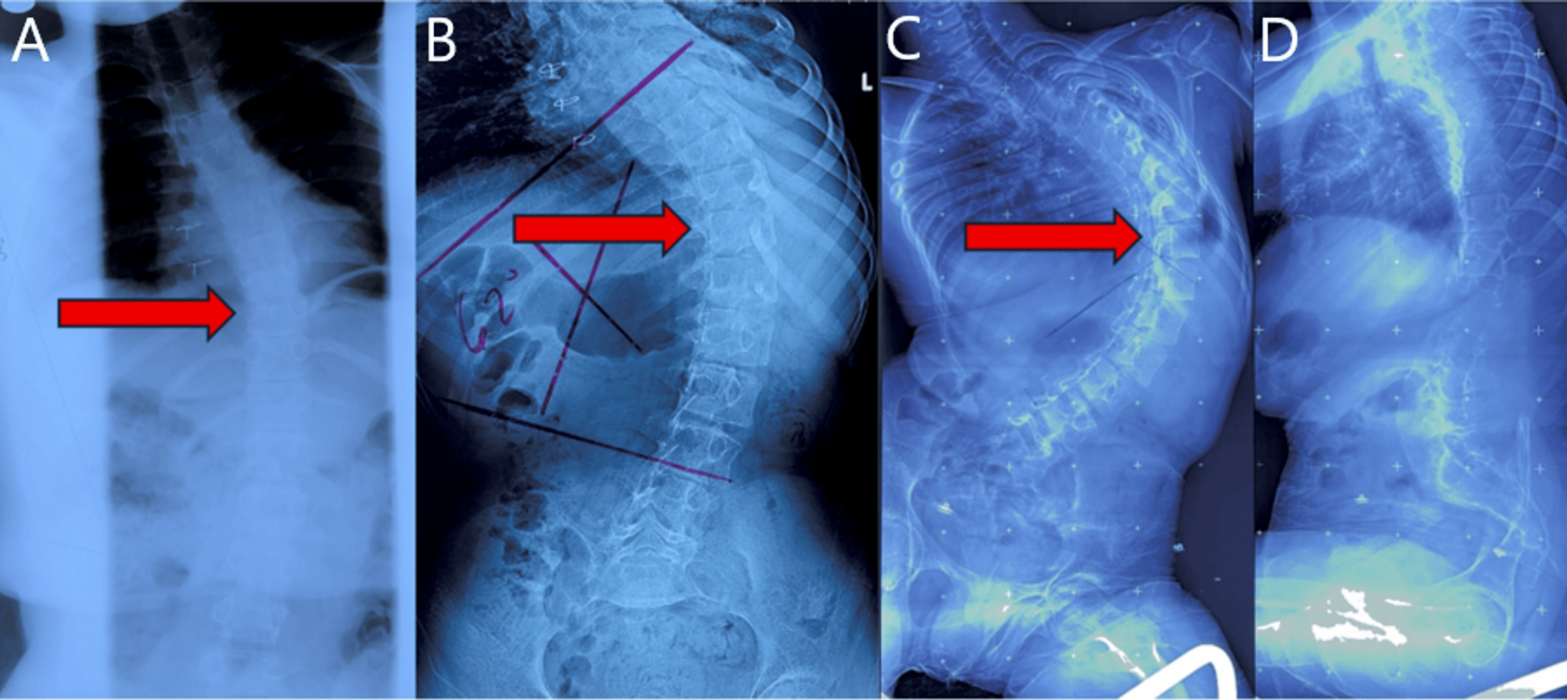
Serial radiographs demonstrating progressive thoracolumbar scoliosis with serial Cobb angle measurements (A) Anteroposterior radiograph at age 13 demonstrating a 33° left thoracolumbar curve measured using the Cobb angle method. (B) Anteroposterior radiograph at age 17 demonstrating progression to a 62° left thoracolumbar curve by Cobb angle measurement. (C) Anteroposterior radiograph at age 18 demonstrating further progression to a 98° left thoracolumbar curve with apex at T10 by Cobb angle measurement. (D) Seated lateral radiograph at age 18 demonstrating a relatively balanced sagittal profile. Arrows indicate the apex of deformity.

Scoliosis was first identified at age 13, measuring 33°, and initially managed with Boston bracing (Figure 1A). Bracing was discontinued after two years following a spontaneous pneumothorax due to concern for compromised pulmonary function. Subsequent radiographs demonstrated curve progression (Figure 1B), prompting referral. At age 18, standing radiographs revealed progression to a 98° thoracolumbar curve (Figure 1C, 1D). Neurologic examination demonstrated hypotonia and neuromotor delay. 

At the time of final evaluation, the patient was considered skeletally mature based on age and radiographic assessment. Musculoskeletal evaluation demonstrated a severe thoracolumbar deformity in the setting of hypotonia and neuromotor delay, without reported congenital spinal malformations on the available imaging. In this context, the scoliosis was considered more likely to reflect a syndromic or neuromuscular component than a congenital vertebral etiology. Cardiopulmonary assessment was limited; although bracing had been discontinued following a spontaneous pneumothorax because of concerns regarding pulmonary compromise, no formal pulmonary function testing was available for review at the time of evaluation, and no chronic ventilatory support requirement was documented. Functionally, the patient remained at baseline status, with independent seated mobility by self-propulsion, preserved usual daily activities, and no reported bowel or bladder dysfunction.

Given his preserved functional status, absence of acute neurologic deficits or bowel/bladder dysfunction, and family concerns regarding postoperative mobility, observation was favored over surgical intervention. This decision followed multidisciplinary evaluation and shared decision-making, as the potential risk of postoperative functional decline was felt to outweigh the anticipated benefit at that time. The patient remains under annual follow-up, with surgical management to be reconsidered should function or quality of life decline.

## Discussion

This case describes severe, progressive scoliosis in an adolescent with partial trisomy 13, with a notable and atypical feature of continued curve progression beyond skeletal maturity. While vertebral anomalies have been reported in Patau syndrome, scoliosis has rarely been described, with limited characterization of its progression or clinical impact [9-11]. To our knowledge, severe scoliosis progression beyond skeletal maturity in an adolescent with partial trisomy 13 has not been well characterized in the available literature, making this a rarely documented presentation that expands the known musculoskeletal phenotype of this condition.

The continued progression of curvature to 98° after skeletal maturity contrasts with the natural history of adolescent idiopathic scoliosis, in which curve progression typically stabilizes following growth completion [12]. In this case, the deformity likely reflects a syndromic scoliosis mechanism rather than a typical idiopathic pattern. In the setting of hypotonia and neuromotor delay, reduced dynamic truncal support may have contributed to progressive coronal imbalance and deformity. Neuromuscular imbalance may also impair the spine’s ability to maintain alignment over time, particularly in the presence of asymmetric loading. More broadly, scoliosis in syndromic conditions may be influenced by abnormal growth modulation, altered biomechanical loading, and, in some cases, connective tissue or structural support abnormalities, which together may contribute to more aggressive and less predictable progression.

Similar patterns of aggressive scoliosis have been reported in other chromosomal abnormalities. Pagadala et al. demonstrated increased prevalence and severity of scoliosis in patients with Turner syndrome, with limited response to bracing and frequent need for surgical intervention [13]. Likewise, Graul et al. reported progressive scoliosis in a patient with partial trisomy 19, who was successfully managed surgically [14]. These reports support an association between chromosomal abnormalities and scoliosis patterns that may differ from adolescent idiopathic scoliosis in both progression and treatment response. More broadly, experience from syndromic scoliosis populations, including neuromuscular and connective tissue-associated disorders, suggests that deformity progression may be less predictable and that conservative measures may be less effective than in idiopathic cases.

The limited effect of bracing in this patient may also reflect features commonly encountered in syndromic scoliosis. Bracing may offer postural support or delay progression in selected patients, but it may fail to reliably control deformity when curve behavior is driven by hypotonia, abnormal biomechanics, or atypical growth patterns rather than the mechanisms seen in idiopathic scoliosis. Although brace compliance was not formally quantified, treatment effectiveness in this setting may also have been influenced by underlying hypotonia, neuromuscular imbalance, and reduced ability to achieve or maintain corrective forces. In addition, bracing was discontinued after a spontaneous pneumothorax because of concern for pulmonary compromise, further limiting the duration and potential effectiveness of conservative management. Surgical correction was considered; however, decision-making was influenced by the patient’s preserved baseline mobility and daily function, absence of acute neurologic deficits or bowel/bladder dysfunction, family concerns regarding postoperative functional decline, and the potential pulmonary and anesthetic risks associated with major surgery in a syndromic patient.

Management of scoliosis in patients with rare genetic syndromes must be individualized, balancing curve magnitude, functional status, comorbidities, perioperative risk, and quality of life. The rarity of partial trisomy 13 limits understanding of scoliosis progression and complicates evidence-based decision-making. No established guidelines currently exist for scoliosis management in partial trisomy 13. This case underscores the importance of longitudinal surveillance and multidisciplinary assessment, particularly when curve behavior diverges from expected idiopathic patterns. Ongoing follow-up is essential, as continued progression may increase the risk of functional decline or neurologic deterioration and alter the balance of risks and benefits of surgical intervention.

## Conclusions

This case highlights severe progressive scoliosis as a rare and underreported manifestation of partial trisomy 13, including continued progression beyond skeletal maturity. It underscores the importance of individualized, multidisciplinary management when treatment decisions must balance deformity progression against functional status and quality of life. Although limited by the single-case design, this report expands the recognized musculoskeletal phenotype of partial trisomy 13 and may provide useful clinical insight for clinicians caring for patients with syndromic scoliosis.
